# Beer-Drinking in Relation to Health and Longevity

**Published:** 1884-06

**Authors:** 


### Beer-Drinking in Relation to Health and Longevity.

There are no vital statistics which can be relied on so confidently as those collected by Insurance Companies; none so free from preconceived conclusions and the bias of personal interest. Statistics collected from this source refer directly to health and longevity; and every interest of the parties collecting them is served by the strictest regard for truth and exactness. We see it stated that the President of the Mutual Life Insurance Company, of Connecticut, one of the oldest and best established companies in the country, has for years been investigating the relation of beer-drinking to longevity, with a view towards determining whether beer-drinkers were desirable subjects for insurance. The result conforms to the opinion long held by medical and other observers. He selected a group of habitual beer-drinkers, and found that, although for two or three years there was nothing remarkable, yet soon afterwards they began to give way, and then the mortality became astounding and uniform. There was no mistaking it; the history was almost invariable; robust, apparent health, full muscles, a fair outside, increasing weight, florid face, then a touch of cold or a sniff of malaria, and instantly some acute disease, with almost invariable typhoid symptoms, was firmly established, and ten days, or less, ended it. He pronounces beer-drinking as peculiarly deceptive at first and throughly destructive at the last. We had precisely the same testimony more than half a century ago, from Sir Astley Cooper, the greatest surgeon of his day. His experience in St. Bartholomew's Hospital led him to declare that the porters and other employes of the London breweries, though of Herculean form and apparently the most robust health, succumbed to diseases with scarcely a show of resistance. The slightest injury often proved fatal to them. They would die, he said, from the scratch of a pin.

